# Comparison between relining of ill-fitted maxillary complete denture versus CAD/CAM milling of new one regarding patient satisfaction, denture retention and adaptation

**DOI:** 10.1186/s12903-024-05298-z

**Published:** 2025-01-04

**Authors:** Maha Nagy Mohamed Kamal

**Affiliations:** Faculty of Dentistry, British University in Egypt (BUE), Shorouk, Egypt

**Keywords:** Relining, Digital denture, CAD/CAM milling, Retention, Denture adaptation

## Abstract

**Purpose:**

This study aimed to compare different treatment modalities to correct ill-fitted maxillary complete denture either by the conventional relining method or by scanning the relining impression and digitally construct a new denture regarding patient satisfaction, denture retention, and adaptation.

**Materials and methods:**

Twelve edentulous patients suffering from loose maxillary complete dentures were selected, dentures’ borders and fitting surfaces were prepared, and relining impressions were taken, the impressions were scanned and the STL files were used for CAD/CAM milling ( computer aided designing/ computer aided manufacturing) of new maxillary dentures (Group A), then the relining impression went through the conventional laboratory steps to fabricate (Group B) maxillary dentures. Both groups were evaluated regarding patient satisfaction by a specially designed questionnaire, retention values were measured by a digital force gauge at denture insertion appointment and two weeks later, geomagic software was used to evaluate dentures adaptation to oral tissues.

**Results:**

Both groups (A and B) were completely satisfied with their dentures except regarding esthetics, all group A and 50% of group B were satisfied. Both groups showed a statistically significant increase in retention values at the two-week follow-up period compared to those at denture insertion time, with higher values were for group B. Finally, the relined dentures showed better oral tissue adaptation than digitally constructed dentures.

**Conclusion:**

Relined maxillary dentures showed better retention, esthetics, and denture adaptation with lower cost than digitally constructed maxillary dentures which showed acceptable retention and adaptation, with better time and data saving.

**Trial registration:**

Clinical trials number: NCT06366321. With registration date on ClinicalTrials.gov public website: 13/ 4/ 2024.

**Supplementary Information:**

The online version contains supplementary material available at 10.1186/s12903-024-05298-z.

## Introduction

It is well known that edentulous ridges progressively diminish in size throughout the patient’s life, especially after complete loss of all natural dentition; consequently, all denture wearers are required to have regular follow-up visits to maintain optimal denture retention and adaptation which reflects on denture efficient serviceability. Denture correction by relining or even rebasing may be considered the most common option of treatment [1].

It was reported that, after relining procedures for ill-fitting dentures, patients expressed a greater level of comfort with their new dentures [2]. On the other hand, as a result of multiple laboratory procedures, material dimension changes, polymerization shrinkage, and residual monomer that may cause pain and mucosal reactions, many edentulous patients are not completely pleased with their dentures and maintain complaining from discomfort, pain, and inadequate retention while using the denture [3]. Patients with compromised medical history that contra-indicate the use of dental implants require to have more valid and reliable treatment options for constructing more satisfactory complete dentures, as a result, providing patients with alternative treatment options and different manufacturing techniques is one of the main goals of merging modern innovations into dental clinics [4].

Digital dentistry plays an extremely important role in removable dentures fabrication recently, it always requires a very accurate impression in order to produce retentive and well adapted denture. To digitally fabricate a prosthesis, a digital impression is obtained either from intraoral scanning of the residual ridge or extra-oral scanning of the master cast and send to the dental laboratory to create a digital model on which the denture is designed and manufactured either by additive or subtractive method [5].

Computer-aided design (CAD) computer-aided manufacturing (CAM) systems prove to reduce complicated laboratory steps, time and effort consumed by the laboratory technicians, the edentulous patients and the dentists [6, 7]. Patient’s data is recorded and stored for future use if duplicate dentures are needed [8]. Digital dentures showed satisfying clinical outcomes with a smoother, more regular surface than conventionally processed ones [9–14]. The digitally fabricated complete denture from resin discs that were produced under elevated temperature and pressure to overcome the problem of dimension changes of heat-polymerized acrylic resin [15].

There were many objective and subjective factors that influence patient satisfaction as age, gender, denture past experience, mastication and chewing ability, appearance, denture retention and adaptation, patient’s pain threshold and the psychological and behavioral manners of the patient [16].

Complete denture retention and adaptation are greatly affected by many factors as method of fabrication, denture extension, shape and compressibility of the supporting tissues, the thickness of the denture base, the material from which denture constructed. The digital superimposition of scanned denture fitting surface with either the scanned oral tissues or a cast is a recently introduced approach to evaluate and compare the adaptation of the constructed dentures [17].

Hence, the method of denture fabrication directly affects its retention and clinical performance, which consequently affects the patient’s satisfaction. A question may arise regarding the effectiveness of incorporating digital technology with the conventional relining technique which may overcome the drawbacks of denture flasking that deform the denture during processing, and to enhance denture retention and adaptation to reach patient satisfaction.

The null hypothesis of this study that, there were no significant differences between digitally and conventionally constructed dentures regarding denture retention and adaptation and patient satisfaction.

## Materials and methods

Twelve patients, both males and females, were selected from the outpatient clinic of the dental faculty, the British University in Egypt (BUE) with complete dentures constructed in the last two years. The patient’s main chief complain was loose ill-fitted maxillary complete denture.

### Inclusion criteria

1. Patients having completely edentulous maxillary and mandibular arches accompanied by normal skeletal relationship (class I) with well-developed maxillary edentulous ridge, and without severe undercuts to minimize the effect of undercuts on retention values.
2. The patients were suffering from a loose or “falling” denture but with proper esthetics, centric relation, and the correct vertical dimension of occlusion (VDO).

### Exclusion criteria

1. Patients with significant skeletal problems (class II or III) or who went through severe osseous surgery or any hereditary or acquired osseous abnormality find it challenging to place the denture properly on the supporting ridge.
2. If the patient suffers from xerostomia, excessive flabby ridges, any pathology of the oral mucosa, or any inflammatory changes, candidiasis, hyperplasia, neurological disorders, and malignancies
3. If the denture creates a major speech problem, poor esthetics, or an unsatisfactory jaw relationship.

The current study has been accepted by the Committee of Ethics, Faculty of Dentistry, British University in Egypt with approval number (23–067). All the participants have been informed about the treatment plan, the follow-up recalls needed and signed written consents.

### Steps for relining the maxillary denture

Tissue preparation: Before making the relining impression, patients were advised to remove their previous dentures from their mouth for at least three to four days and massage their soft tissues two to three times per day to simulate blood flow and improve the recovery of any small ulcers or inflammations with the application of proper medicaments.

Denture preparation: Occlusion must be balanced before the relining process to guarantee even occlusal contact and that the denture will not move or tilt when the patient is asked to close his mouth during impression material setting. To prevent contact with the reflected tissues, denture borders were lowered by around 2 mm, except the denture posterior border (post dam). The denture tissue surface was relieved by about 1–2 mm to create even space for impression material.

Border molding with putty consistency rubber base impression material (Speedex addition silicon, Coltene, Switzerland) was done, then the denture fitting surface was loaded with light body rubber base impression material, which should flow evenly to cover the entire fitting surface and the borders of the denture with a thin continuous layer. The denture was then inserted inside the patient's mouth, and border molding was completed manually and functionally.

**Fig. 1:**
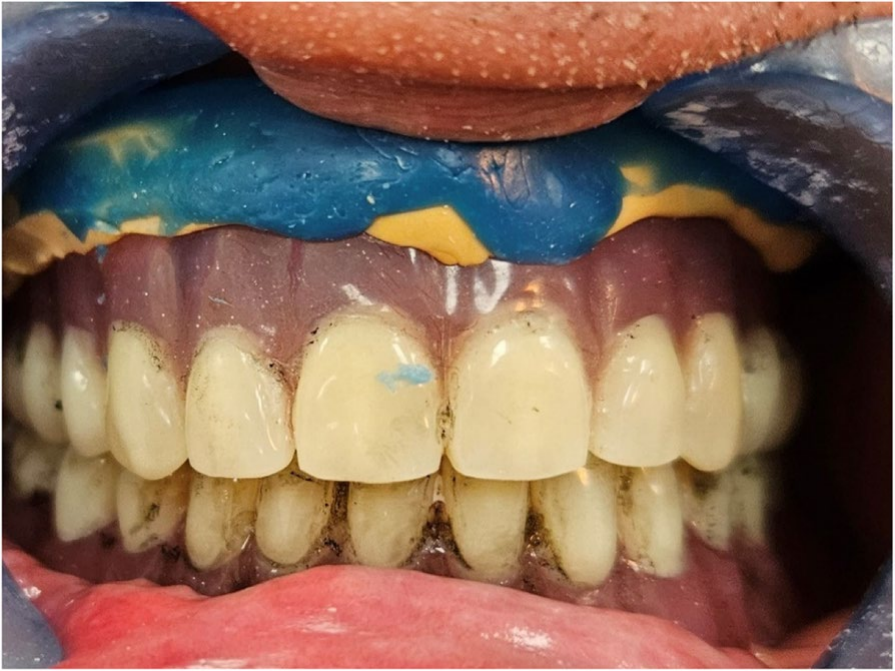
Patient closed in centric while relining impression setting

To stabilize the upper denture throughout the impression material setting process, patients were advised to close their mouth, and that therefore a "closed mouth reline protocol" was followed. (Fig. 1) After the material setting, the denture was taken out of the patient’s mouth, and the impression was examined for any imperfections and should show an accurate reproduction of the denture-bearing area.

### The relining impression was used to construct a new maxillary denture by either digital or conventional technique

#### Digital technique of denture construction

**Fig. 2:**
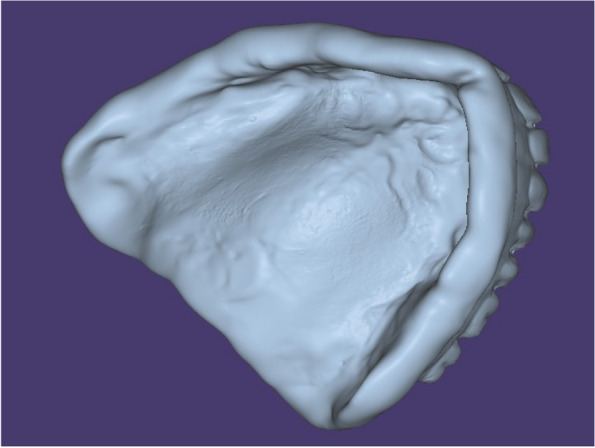
Scanned maxillary denture with relining impression

**Fig. 3:**
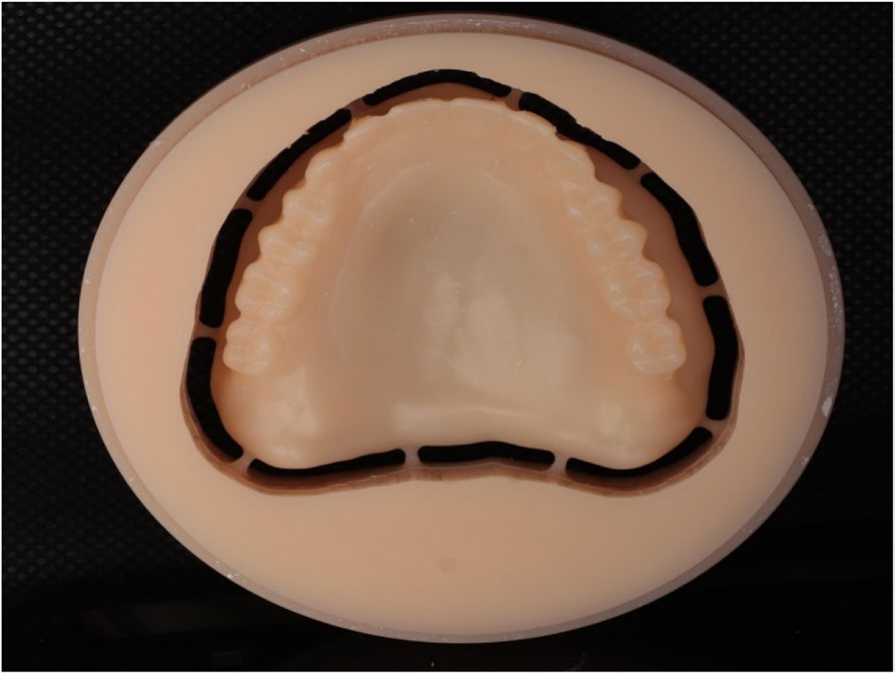
CAD/CAM milled denture

**Fig. 4:**
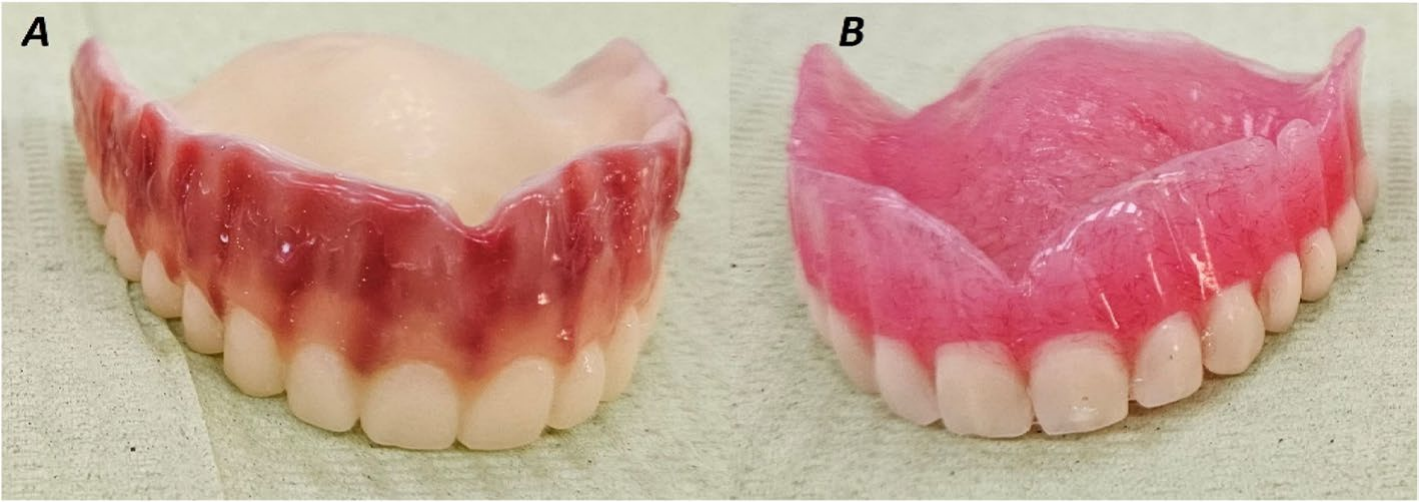
**A** Finished milled denture, **B** Finished relined denture

Before boxing and pouring the relining impression, the maxillary denture was scanned from all surfaces, fitting (impression surface), polished, and occlusal surfaces using a desktop scanner (3Shape E2, Copenhagen, Denmark.) the scanned images were exported to the computer software as standard tessellation language file (STL) (Fig. 2). STL file was imported into a dental CAD/CAM milling machine software program (Exocad DentalCAD; exocad, GmbH, Germany) to fabricate a new maxillary complete denture after finalizing the denture design digitally from PMMA discs of 25 mm length (Yamahatchi dental MGF, Japan) (Fig. 3). After milling, finishing, denture characterization were completed using three shades of composite. (Visio,lign, Bredent, GmbH, Germany) (Fig. 4A).

#### Conventional technique of denture construction

After boxing the impression, an extra hard stone material (Gypsum type VI, BMS, Italy) was used for impression pouring making sure that any extra material on the base's teeth or facial surfaces was eliminated. The denture and the cast shouldn't be separated.

Denture processing was completed by flasking the denture into a metal flask, immersed it into boiling water for wax elimination, packing the heat cure acrylic resin (Acrostone, Egypt), curing the resin by long cycle in a hot water bath at 72 ◦C for 6.5 h, then deflasking, finished and polished was done [17] (Fig. 4B).

#### Sample size calculation

A pilot study was conducted measuring retention and adaptation of 5 samples of the relined dentures group and the CAD/CAM milled dentures group. The Cohen’s d effect size was measured from the pilot study. Based on the Cohen’s d effect size with assuming a type I error of 0.05 and a study power of 0.9, the sample size required to detect a significant difference between the two groups was increased to include 12 samples.

#### Patient’s randomization

This study was designed to be a crossover study were the twelve patients had received one denture from both groups, a block randomization was done by the author to determine the order of dentures insertion (which denture group to deliver first).

#### Denture delivery

Dentures were delivered after examination of fitting and polished surfaces for any nodules or sharpness, occlusal adjustments were done if needed by selective grinding. Post-insertion instructions were the same as given for any removable prosthesis. After the end of the two weeks follow up period of the first group of dentures, a resting period of one week was given to all patients before delivering and evaluating the other group of dentures.

Randomization and resting period were needed to avoid the effect of the order of the prosthesis on the measurements of denture retention and evert the influence of muscle adaptation on the obtained results.

Data were collected at the appointment of denture insertion and two weeks later and used to evaluate patient satisfaction, denture retention, and adaptation.

I. Patients’ satisfaction evaluation through a questionnaire

The questionnaire used was developed especially for this study and designed as a version of the Likert scale with ranges from 1 (completely unsatisfied) to 5 (completely satisfied), representing the denture satisfaction scale at denture insertion time and two weeks later regarding denture retention, mastication, speech, and esthetics [18].

Example of the questionnaire for patient satisfaction measurement.

- On a scale from 1 to 5, how do you feel about your denture retention?

**Table:** 

**At insertion time**				
**1**	**2**	**3**	**4**	**5**
**Completely unsatisfied**		**Satisfied**		**Completely satisfied**
**Two weeks later**				
**1**	**2**	**3**	**4**	**5**
**Completely unsatisfied**		**Satisfied**		**Completely satisfied**

The questions were repeated regarding mastication, speech, and esthetics.

Patient-based outcomes data were collected. A total satisfaction score was calculated for each item; higher scores mean greater satisfaction.

II. Denture retention evaluation

**Fig. 5:**
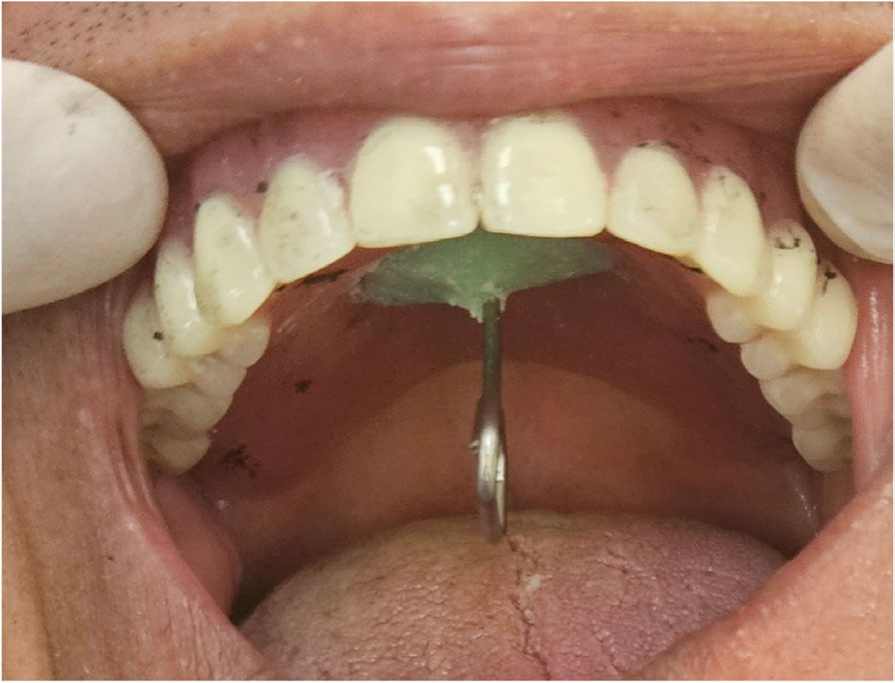
Denture with the hook seated intraoral

A (19-gauge) orthodontic wire in the form of a hook was secured on the center of the palate at a point of intersection of the lines connecting the hamular notches, the right and left canines with the midline by self-cured acrylic resin material (Acrostone, Egypt), then the denture placed on the supporting tissues intraorally (Fig. 5).

### Testing procedure

**Fig. 6:**
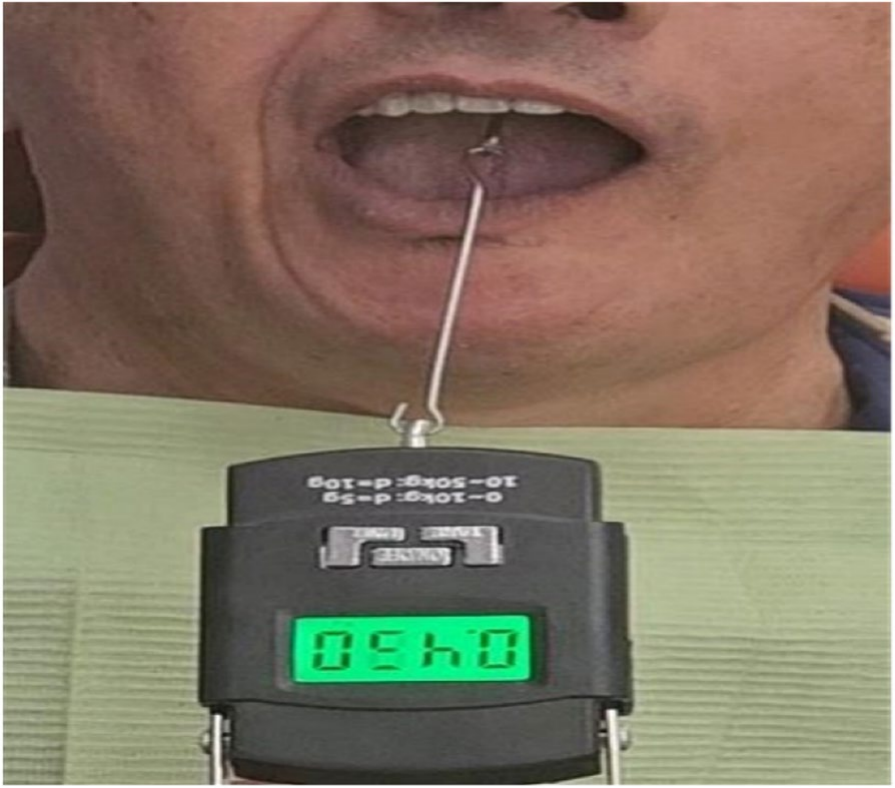
Retention measurement

The patient was in an upright position. A digital force gauge (Force Gauge HF- 50N) was linked to the hook and a downward pulled force was applied slowly in a vertical direction opposite to that of the denture path of insertion until the denture was displaced, the force value recorded at the time denture displacement was registered. After repeating this procedure three times, the average was determined (Fig. 6) [1, 19, 20].

I. Denture adaptation evaluation

Maxillary arches of all patients were scanned using an intraoral scanner (3 shape TRIOS 4 wired, Copenhagen, Denmark), fitting surfaces of both denture groups were also scanned using desktop scanner ( 3Shape E2, Copenhagen, Denmark).

**Fig. 7:**
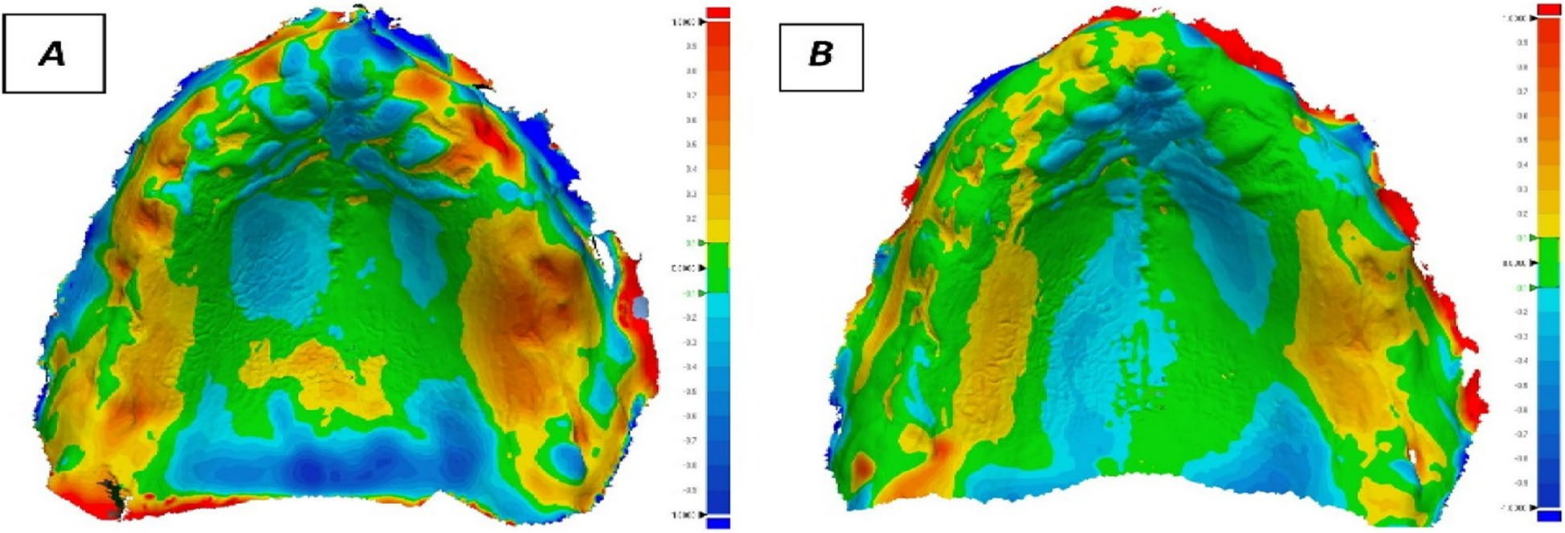
**A** Color map of group A adaptation. **B** Color map of group B adaptation. Pressure from the denture fitting surface to the oral tissues is indicated by yellow to red colors. The denture fitting surface and oral tissues are separated by a blue color. The green color denotes that the oral tissues and the denture fitting surface are in contact

After scanning of the denture fitting surface, the STL file was imported to a three-D measurement program (Geomagic Control X, 3D Systems, United States) software to flip the fitting surface of the dentures to resemble the intra-oral tissue surface to build and superimpose STL files using first initial alignment and then best-fit alignment (Fig. 7A and B). For every measurement point, the root mean square (RMS) (mm) was computed considering both positive and negative-values, The adaptation deviations of the repaired dentures were calculated as done by the previous study [21].

Statistical analysis for retention values and data was tested for normality using the Shapiro–Wilk test. Between-group comparisons were conducted using an independent* t* test, while within-group comparisons were conducted using paired *t* test. The significance level was set at *p* = 0.05. statistical analysis was completed by SPSS software (version 25). While statistical analysis for adaptation, data descriptive statistics consisted of mean, standard deviation, median, minimum, and maximum values. Data was tested for normality using the Shapiro–Wilk test and an independent *t* test was used for between-group comparison. The significance level was set at 0.05. Statistical analysis was completed by SPSS software (version 25).

## Results

### Patient satisfaction results

All patients of both groups A &B were satisfied with their dentures regarding retention, chewing ability and speech. All the patients of group B (relined dentures) and half the patients (50%) of group A (digitally constructed dentures) were satisfied regarding denture appearance and esthetics.

### Retention results

**Fig. 8:**
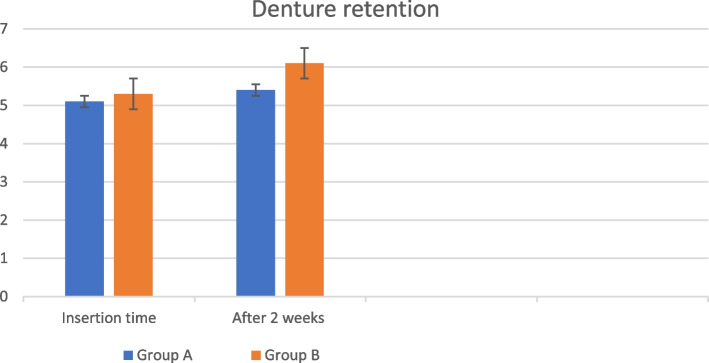
Bar chart representing the retention values of both groups at denture insertion time and two weeks after

Retention values were significantly higher after two weeks than at the insertion time (*p* < 0.001) within both groups. Group B showed significantly higher retention than group A at the insertion time (*p* = 0.007) and 2 weeks later (*p* < 0.001). As showed in Table 1 Fig. 8.

**Table 1:** Descriptive statistics, the results of independent *t* test in the rows for between-group comparison and the results of paired ***t*** test in the columns for within group comparison at different time intervals

		**Group A**	**Group B**	***p-*value**
**Insertion Time**	**Mean (SD)**	5.0 (0.2)	5.2 (0.2)	0.007*
	**Median (Range)**	5.1 (4.7—5.3)	5.3 (5.0—5.4)	
**After 2 weeks**	**Mean (SD)**	5.5 (0.1)	6.1 (0.2)	< 0.001*
	**Median (Range)**	5.4 (5.3—5.7)	6.1 (5.8—6.4)	
***p-*value**		< 0.001*	< 0.001*

### Denture adaptation results

**Fig. 9:**
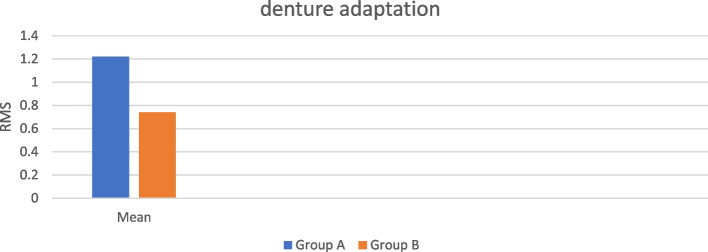
Bar chart representing the mean RMS in both groups

Group A showed significantly higher RMS (less adaptation) than group B (*p* < 0.001) with a mean difference 0.47 and 95%CI: (0.46,0.48). as showed in Table 2 Fig. 9.

**Table 2:** Comparison between the results of independent *t* test of RMS values of the two groups

Descriptives	Milled Group	Relined Group	*p-*value
*Mean*	1.22	0.74	< 0.001*
*SD*	0.01	0.01	
*Median*	1.22	0.75	
*Min*	1.20	0.72	
*Max*	1.24	0.76

## Discussion

The success of any prosthesis in terms of speech, function, appearance, and denture retention is thought to be greatly influenced by patient happiness. The primary factor considered when assessing the clinical performance of complete dentures is their ability to remain in the mouth during function. Wearers of CDs always have one basic complain: insufficient retention [22]. The current in-vivo study was conducted to assess the degree of patients’ satisfaction and compare the adaptation and retention gained from relining loose maxillary denture conventionally using flasking procedure versus a newly CAD/CAM milled maxillary denture obtained from scanning the relining impression to fabricate complete denture digitally.

Many parameters, including physical factors (adhesion, cohesion, surface tension and peripheral seal) and mechanical factors (mechanical locking into undercuts and ridge anatomy), have a significant impact on the retention of complete maxillary dentures [20]. Therefore, patients with xerostomia, viscous saliva and/or severe bony undercuts were excluded from this study.

In this study, closed mouth impression technique of relining was carried out. The main point of creating a suction-effective denture is completely sealing the entire border of oral mucosa to prevent air invasion so negative pressure will be established [23].

The current study findings stated that all patients were satisfied with both of their dentures regarding retention, speech and chewing ability (occlusion) this is mainly because the digital dentures were milled from the scanned old denture with the same teeth setting and relationship, so the occlusion and speech weren’t changed. On the contrary, Kang YJ et al. [24] did a cross-over single-blinded randomized in-vivo study to compare between the digitally fabricated complete dentures versus conventionally fabricated complete dentures. It was found that masticatory efficiency and speech with the digitally fabricated complete dentures were less favorable than the conventionally fabricated complete dentures, however, comparable results were obtained for denture adaptation and patient satisfaction between the two denture groups.

Saponaro et al. had studied and compared patient satisfaction of digitally constructed dentures and it was shown that, 3 (6.25%) of the 48 participants reported having bad esthetic from their CDs, while 1 of the 48 individuals (2.08%) suffered from altered phonetics. On the second visit, 24.44% of the digital dentures were not placed because of poor retention, an improper centric relationship, poor aesthetics, and altered phonetics [25].

At the present study, retention values were recorded at insertion time and two weeks after, no additional follow up periods were needed as it was claimed that no significant changes in retention values between conventional dentures and digital dentures were recorded for longer follow up periods than 2 weeks [26].

Group B (relined dentures) showed a statistically- significant higher retention values at all follow up periods and also more denture adaptation compared to group A (milled dentures), this may be explained by the fact that relining impression was taken under patient’s biting force, so it is muco-compressive impression recorded the areas of the peripheral soft tissues under compression. The same outcome was revealed from an in-vivo study comparing digital and conventional impression technique in edentulous maxillae and it was revealed that conventional impression-taking is more precise than digital impressions in recording the peripheral soft tissues responsible for creating strong peripheral seal areas to ensure retentive removable prostheses [27].

Over-extended borders of digitally constructed maxillary dentures was found to be another reason that could explain their reduced retentive values compared to conventionally constructed dentures as shown in a study done to compare the digitally and the conventionally constructed complete denture, it was claimed that over-extended borders of the digital dentures were obvious at the time of denture insertion caused denture displacement and reducing the retention of digital denture, however, after two weeks and three months follow up periods, no significant differences were found between both denture groups [28]. However, in this study overextended denture borders was minimal and not considered the main cause of reduced retention of digitally constructed dentures.

Many authors stated that the larger the scanned distance along the edentulous arch the more the horizontal error occurred [29]. This may be due to a progressive error acumination while producing the final image from collecting and translating single separate images. The wider areas of the edentulous arch are captured the more numbers of images would be superimposed, so the final data will be distorted as a result of the inaccuracies accumulation generated. On the other hand, laboratory (desktop) scanners don’t show this similar pattern of deviation [30].

The scanner employed in this study was able to accurately scan the borders of the existing dentures, occlusal surface, and the relining impression surface (fitting surface) of the old denture with precise capturing of complete denture contours and soft tissue details [31, 32]. however, the fitting surface of the milled denture revealed a highly smooth texture that hinders improved appropriate retention, border seal, and physical adaptability to the underlying tissues. Another study evaluated denture retention of digitally constructed dentures (milled and printed). Retention values of both denture groups after 2 weeks follow up period were significantly improved from baseline, the reason for this might be related to the settling of the denture base, functional adaptation, and neuromuscular coordination of the patients [10].

It should be known that there is no intraoral scanner could produce a true precise functional impression with proper borders extension and shape. Nonetheless, without functional molding of the vestibular mucosa and slight compression on the highly-resilient posterior palatal seal areas, an acceptable border and palatal seal wouldn’t be achieved, which consequently, affect denture retention negatively [33]. With the same concept, a study was done to compare the accuracy of scanning edentulous maxillary arch using different types of intraoral scanners versus scanning the conventional impression and the referring casts (laboratory scanners) and the results showed that laboratory scanning revealed more accurate results [34].

Other adverse outcomes reported with digital complete dentures regarding differences in VDO measurements, poor esthetics, bad teeth setting, and impaired speech [35]. In the present study, patients had their existing loose dentures constructed from 2 years or less with proper VDO and correct centric relation [36, 37] to avoid teeth wear, low VDO and to ensure proper occlusion for the digitally constructed denture.

Moreover, many other studies confirmed that milling burs size, shape, and sharpness played a significant role in the shape of the final milled prothesis regarding its surface roughness and surface adaptation which limits the accurate reproduction of denture fitting surface details that will negatively affect the intimate adaptation with oral mucosa [38, 39].

However, in pilot cohort research done by Bidra et al., it was found that while patients expressed great satisfaction with phonetics and esthetics, just 50% of them were dissatisfied with the retention, stability, or adaptability of their digitally produced dentures [40]. In contrast, Kattadiyil et al. [41] reported no discernible change in phonetics or esthetics and discovered a markedly higher retention rate for milled maxillary complete dentures when compared with conventional ones made for the same patient. Patients who had digital dentures scored better overall, and for denture base contour, fit, stability, retention, and extension. This was explained by the fact that, in contrast to traditional ones, there was no polymerization shrinkage throughout the fabrication process, which resulted in greater fit and retention [42–44].

A prospective cross-over study on clinical effectiveness and patient satisfaction with digital dentures against conventional dentures was conducted by other studies. They found that while the method of manufacture had no effect on patient satisfaction, there was a tendency for digital dentures to be more clinically successful than traditional dentures [45, 46], which was not proved in this study.

## Conclusion

Within the limitation of this study, it could be concluded that:

- Ill-fitted maxillary complete dentures corrected by conventional relining procedure showed better esthetics, retention, and adaptation than digitally constructed maxillary dentures which showed acceptable esthetics, retention, and adaptation with better time and data saving.

### Clinical impact

The idea of incorporating relining impression of loose old denture with scanning, designing and fabricating complete dentures digitally is reliable and could result in successful denture. although this digital manufacturing method could not improve denture retention and adaptation compared to the conventional relining procedure, but it has the advantages of avoiding material discrepancies during flasking and curing, in addition to avoiding complicated laboratory steps and long processing time.

### Limitations and future prospectives

This study has been tried to replace conventional laboratory steps of denture relining with more digital, easy, and time saving ones, but some limitations were observed; considering that intraoral scanners are reliable devices for imaging oral tissues with great accuracy, however, recording the tissues with slight pressure is not achieved till now. Future studies are recommended to investigate the possibility of substituting the conventional clinical steps of denture construction with digital ones to convert prolonged clinical appointments to be less time consuming and more reliable with analysis related to the functional effectiveness of the resultant dentures should be considered.

## Supplementary Information

Supplementary Material 1.

## Data Availability

No data availability, there are no data underlying the manuscript, all data were presented by figures and tables at the current study.

## Abbreviations

CAD/CAM: Computer aided designing/ computer aided manufacturing
VDO: Vertical dimension of occlusion
CDs: Complete dentures
