# Supplementary material for: Comparison between relining of ill-fitted maxillary complete denture versus CAD/CAM milling of new one regarding patient satisfaction, denture retention and adaptation
Source: BMC Oral Health. 2025 Jan 4;25:18. doi: 10.1186/s12903-024-05298-z (PMC11699795; doi:10.1186/s12903-024-05298-z)
Supplement: Supplementary file 1 — Supplementary Material 1. [file 12903_2024_5298_MOESM1_ESM.pdf]

Patient name ………………….. ……………………………. ………………………. Denture group ………..

Questionnaire for patient`s satisfaction measurement.

- **On a scale from 1 to 5, how do you feel about your denture retention?**

At insertion time

1	2	3	4	5

Completely unsatisfied Satisfied Completely satisfied

Two weeks later

1	2	3	4	5

Completely unsatisfied Satisfied Completely satisfied

- **On a scale from 1 to 5, how do you feel about your mastication ability?**

At insertion time

1	2	3	4	5

Completely unsatisfied Satisfied Completely satisfied

Two weeks later

1	2	3	4	5

Completely unsatisfied Satisfied Completely satisfied

- **On a scale from 1 to 5, how do you feel about your speech improvement?**

At insertion time

1	2	3	4	5

Completely unsatisfied Satisfied Completely satisfied

Two weeks later

1	2	3	4	5

Completely unsatisfied Satisfied Completely satisfied

- **On a scale from 1 to 5, how do you feel about your esthetics improvement?**

At insertion time

1	2	3	4	5

Completely unsatisfied Satisfied Completely satisfied

Two weeks later

1	2	3	4	5

Completely unsatisfied Satisfied Completely satisfied
